# TOP2A deficiency leads to human recurrent spontaneous abortion and growth retardation of mouse pre-implantation embryos

**DOI:** 10.1186/s10020-022-00592-4

**Published:** 2022-12-30

**Authors:** Yuhan Duan, Huijia Fu, Jiayu Huang, Nanlin Yin, Linhong Liu, Xiru Liu

**Affiliations:** 1 Reproductive Medicine Center, The First Affiliated Hospital of Chongqing Medical University, No. 1 Youyi Road, Yuzhong District, 400016 Chongqing, China; 2 Chongqing Key Laboratory of Translational Medicine in Major Metabolic Diseases, Chongqing Medical University, Chongqing, 400016 China

**Keywords:** TOP2A, Proliferation, Apoptosis, Cell cycle, Migration, Invasion, Pre-implantation embryos, Oxidative stress, FOXO signalling pathway

## Abstract

**Background:**

Recurrent spontaneous abortion (RSA), is a dangerous pregnancy-related condition and is a subject of debate in the gynaecology and obstetrics communities. The objective of this study was to determine the function of DNA Topoisomerase II Alpha (TOP2A) in RSA and elucidate the underlying molecular mechanisms.

**Methods:**

In vitro models of TOP2A-knockdown and -overexpression were generated by transfecting specific sh-RNA lentivirus and overexpression plasmid, respectively. An in vitro TOP2A inhibition model was established by culturing mouse embryos at the two-cell stage in a medium containing PluriSIn2, a TOP2A inhibitor. Immunohistochemical staining was used to analyse expression of TOP2A in villi tissues of patients with RSA. Western blotting and qRT-PCR were used to analyse the expression of TOP2A and proteins involved in trophoblast functions, the FOXO signalling pathway, and the development of pre-implantation embryos. 5-Ethynyl-2′-deoxyuridine staining, TUNEL assay and flow cytometry were used to further evaluate the effect of TOP2A on cell proliferation and apoptosis. Transwell and wound healing assays were used to evaluate migration and invasion. Moreover, the effect of TOP2A inhibitor on embryos was determined by immunofluorescence and mitochondrial-related dyes.

**Results:**

Evaluation of clinical samples revealed that the villi tissues of patients that have experienced RSA had lower TOP2A expression compared with that from women who have experienced normal pregnancy (*P* < *0.01*). In vitro, TOP2A knockdown decreased the proliferation, migration, and invasion of trophoblast cell lines, and increased apoptosis and activation of the FOXO signalling pathway (*P* < *0.05*). Conversely, TOP2A overexpression reversed these effects. Moreover, in vivo experiments confirmed that inhibition of TOP2A impairs trophectoderm differentiation, embryonic mitochondrial function as well as the developmental rate; however, no differences were noted in the expression of zygotic genome activation-related genes.

**Conclusions:**

Collectively, our data suggest that lower TOP2A expression is related to RSA as it inhibits trophoblast cell proliferation, migration, and invasion by activation of the FOXO signalling pathway. Additionally, TOP2A inhibition resulted in impaired development of pre-implantation embryos in mice, which could be attributed to excessive oxidative stress.

**Graphical Abstract:**

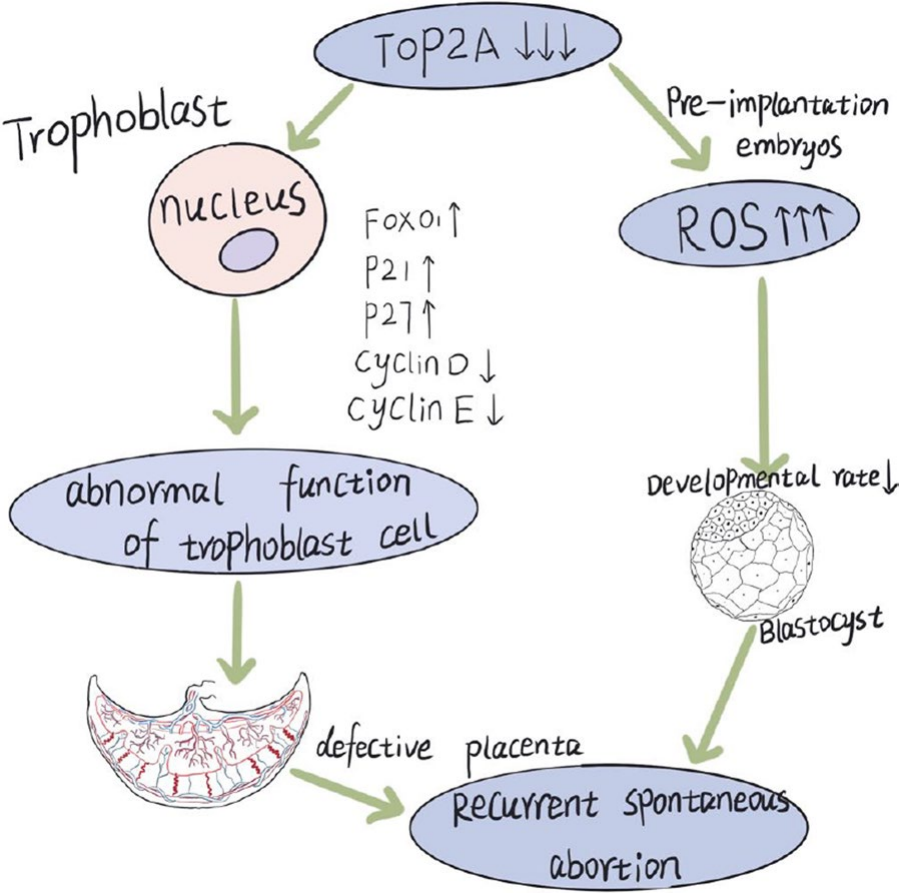

**Supplementary Information:**

The online version contains supplementary material available at 10.1186/s10020-022-00592-4.

## Introduction

According to the American Society of Reproductive Medicine, spontaneous abortion rates range from 15–20%, with recurrent spontaneous abortion (RSA) accounting for roughly 2–5% of all pregnancies (Zhou et al. 2021a). It is a destressing pregnancy-related condition that is experienced by 1–5% of women of childbearing age and is a challenge in gynaecology and obstetrics communities. Recurrent spontaneous abortion, which includes embryonic and foetal losses, is characterised as the failure of two or more clinically consecutive pregnancies before 20–24 weeks of gestation (Dimitriadis et al. 2020). The aetiology of RSA is a multifactorial process that includes abnormal immunological dysfunction, abnormal reproductive structure, abnormal chromosome structure and function, maternal factors, prethrombotic state, and environmental variables (Hachem et al. 2017). Even with all the advancements in the scientific community, the causes of RSA remain largely unknown; therefore, studies that aim to elucidate the underlying causes of RSA are crucial.

Implantation, decidualisation, placentation, and parturition are some of the discrete events that make up the complicated process of pregnancy (Cha et al. 2012). Each event is dependent on the other for the progression of pregnancy. For implantation to take place, an interaction between the embryo and a receptive uterus must be facilitated by molecular and physiological processes. The hierarchical process of embryo implantation necessitates fundamental techniques including apposition, adhesion, attachment, and penetration, in which villous trophoblasts and high-quality embryos play a vital role. During embryo implantation, extravillous trophoblasts (EVTs) of the decidua basalis originating from trophoblastic cell columns of anchoring villi invade into the maternal uterine decidua, up to the inner third of the myometrium. This process, i.e., invasion of EVTs, facilitates the attachment of the placenta to the uterus (interstitial invasion) to provide nutrients to the embryo from the placenta (Moser et al. 2017). Therefore, trophoblasts are the precursor cells of the placenta, and are critical for a successful pregnancy. Previous studies have revealed that defective functions of trophoblasts may be attributed to shallow placentation and abnormal remodelling of the spiral artery, which is subsequently associated with bad pregnancy outcomes, such as abortion and stillbirth.

DNA Topoisomerase II Alpha (*TOP2A*) is a key gene that is implicated in repeated implantation failure (RIF) (Fu et al. 2022). However, the effects of TOP2A on trophoblasts and embryo quality have not been explored. TOP2A is a nuclear enzyme that can regulate and modify the topologic states of DNA during transcription. It facilitates chromatid separation, chromosome condensation, and decreased torsional stress during transcription and DNA replication. TOP2A can wind and unwind DNA strands, allowing the strands to travel past one another and changing the architecture of DNA. Additionally, TOP2A is associated with cell invasion, migration as well as cell cycle. Moreover, it is a vital biomarker for cancers as TOP2A overexpression is associated with several cancers, such as hepatocellular carcinoma (HCC), malignant peripheral nerve sheath tumours, hepatoblastomas, oesophageal cancer, breast cancer, gastroesophageal carcinoma, colorectal carcinoma, pancreatic cancer, and prostate cancer (Heestand et al. 2017; Hooks et al. 2018; Kolberg et al. 2015; Pei et al. 2018). However, the biological role of TOP2A in trophoblasts and consequently in the occurrence and progression of RSA remains unclear. Therefore, in the present study, we aimed to investigate whether TOP2A induces a change in trophoblast functions in terms of proliferation, migration, invasion, and apoptosis and further contributes to RSA. In addition, we used mouse pre-implantation embryos explore whether TOP2A exerts effects on the development and quality of early embryos, such that it can affect implantation and consequently cause RSA.

## Methods

### Patients and tissue samples

All recruited participants were outpatients at the Department of Obstetrics and Gynaecology, The First Affiliated Hospital of Chongqing Medical University. The control group, consisted of placental villi obtained from fifteen fertile women aged 18–38 years with one or more children who made an optional decision to terminate their pregnancy for non-pathological reasons. The elective terminations were conducted during the first-term. None of these women had a history of spontaneous abortion, preterm delivery or stillbirth. On the other hand, the RSA group included fifteen patients aged 18–38 years who had experienced at least twoconsecutive first-trimester losses of unexplained aetiology between 2021 and 2022. Subjects with known risk factors for RSA, such as uterine malformation, hormonal disorder, major chromosomal aberrations, and infectious diseases were excluded in this study. All characteristics of the study participants are shown in Table 1. In the present study, chorionic villous samples were obtained by suction curettage performed according to standard procedures. Following which, fresh villi tissues excluding the bleeding and calcification regions were collected within 10 min after dilatation and curettage, and were kept on ice and transported immediately to the laboratory. A portion of each sample was cut, fixed in 4% paraformaldehyde, dehydrated using gradient ethanol solutions, and embedded in paraffin. This study was approved by the Institutional Ethics Committee of The First Affiliated Hospital of Chongqing Medical University approved the procedures of this research and was conducted in compliance with the Ethical Review Methods for Biomedical Research involving Humans adopted by the National Health and Family Planning Commission of the People's Republic of China.

**Table 1:** Characteristics of the two groups ‘women

	n	Age (years, mean ± sem)	Gestational age(weeks, mean ± sem)	Gravidity (frequency, mean ± sem)	Parity (frequency, mean ± sem)	Number of abortion (frequency, mean ± sem)
Control	15	32.60 ± 1.24	7.35 ± 0.24	3.00 ± 0.40	1.60 ± 0.24	0.53 ± 0.17
RSA	15	29.93 ± 1.23	7.08 ± 0.37	2.67 ± 0.27	0.93 ± 0.21	1.47 ± 0.13
*P*		0.137	0.533	0.497	0.042	< 0.001

*RSA* recurrent spontaneous abortion, *sem* standard error of the mean

### Western blotting

RIPA Lysis buffer (Beyotime Biotechnology, Shanghai, China) supplemented with a phosphatase inhibitor and protease cocktail (Solarbio, Beijing, China) was used for whole protein extraction. BCA protein assay kits (Beyotime Biotechnology) was used to measure protein concentrations. RIPA, 5% dithiothreitol, and loading buffer were used to adjust protein concentrations. Equal amounts of protein (30 μg/lane) were separated using SDS–PAGE and transferred on to PVDF membranes (Millipore Sigma, Burlington, MA, USA), which was subsequently blocked with quick blocking buffer (NCM Biotech, Suzhou, China) for 15 min. Membranes were washed with phosphate-buffered saline (PBS) and then incubated with the following primary antibodies overnight at 4 °C: TOP2A (1:10,000; Abcam, Cambridge, UK), matrix metalloproteinase 9 (MMP9) (1:1000; Abcam), MMP2 (1:1000; Abcam), cyclin-dependent kinase 2 (CDK2) (1:1000, Cell Signaling Technology, Danvers, MA, USA), CDK4 (1:1000, Cell Signaling Technology), phosphor-FoxO1 (1:1000, Cell Signaling Technology), P21 (1:1000; Cell Signaling Technology), P27 (1:1000; Cell Signaling Technology), cleaved caspase 3 (1:1000; Abcam), caspase 3 (1:1000; Abcam), and β-actin (1:2000; Proteintech, Wuhan, China). The membranes were then incubated with horseradish peroxidase conjugated secondary antibodies (1:5000, ZSGB-BIO, Shanghai, China) at room temperature for 1 h. An improved chemiluminescent reagent (Millipore Sigma) was used for visualisation of protein blots, and images were taken using Fusion FX5 Spectra imaging system (Vilber Lourmat, Collégien, France).

### Immunohistochemistry (IHC) analyses

Villi tissue samples were fixed with formalin, and embedded in paraffin. The paraffin blocks were sliced into 3 μm sections. The sections were deparaffinised and re-hydrated by soaking in dimethylbenzene and ethyl alcohol, respectively. Thereafter, antigen retrieval was performed by a microwaved pre-treatment in 10 mM citric sodium (pH: 6.0) for 10 min. Following which, 3% H2O2 was used for blockage of endogenous peroxidase. Thereafter, the slides were incubated with anti-TOP2A antibody (1:1000; Abcam) overnight at 4 °C and then with an appropriate biotin-conjugated secondary antibody. The entire field of vision on the slides were imaged with a Pannoramic SCAN II digital microscope (3DHISTECH, Budapest, Hungary).

We used ImageJ to set the baseline for measuring fluorescence signal in advance to exclude cells with low fluorescence intensity due to fluorescence crosstalk. ImageJ software (National Institutes of Health, Bethesda, MD, USA) was used to measure the average optical density of positive signals per field.

### Cell culture

The immortalised human EVT cell line HTR-8/SVneo and the human choriocarcinoma cell line JEG-3 was kindly provided by Chao Tong from State Key Laboratory of Maternal and Foetal Medicine of Chongqing Municipality. HTR-8/SVneo and JEG-3 were cultured in RPMI 1640 (Gibco, Waltham, MA, USA) and DMEM/F-12 medium (Gibco), respectively, supplemented with 10% foetal bovine serum (FBS) (PAN). Furthermore, the human umbilical vein endothelial cell line Huvec was obtained from Shanghai Cell Bank (Shanghai, China) and cultured in DMEM medium (Gibco). All cells were cultured in a humidified atmosphere with 5% CO_2_ at 37 °C.

### 5-ethynyl‐2′-deoxyuridine (EdU) incorporation assay

To evaluate DNA replication and further assess cell proliferation, EdU assay kits (RiboBio, Guangzhou, China) were used. Cells in logarithmic growth phase were seeded in 96-well plates (0.8 × 10^4^ cells/well) and cultured in DMEM/F-12 medium (Gibco) containing 10% FBS for 24 h prior to the addition of the EdU reagent. The supernatant was discarded and subsequently cells were incubated with diluted EdU (50 μmol/L) for 2 h. Next, cells were fixed with 4% formaldehyde and permeabilised with 0.5% TritonX‐100 for 10 min at 25 °C. The cells were then stained with the fluorescent dyes containing EdU and Hoechst as per the manufacturer’s instructions (RiboBio). Fluorescence microscopy (ZEISS, Oberkochen, Germany) was used to capture images.

### TUNEL assay

One step TUNEL apoptosis assay kits were purchased from Beyotime Biotechnology. As per the manufacturer’s instructions, the treated cells were seeded in a 12-well plate with equal densities. After 24 h, the cells were washed, fixed, permeabilised, and incubated. Finally, images were captured using an inverted fluorescence microscope (ZEISS).

### Flow cytometry

For apoptosis analysis, first, the total medium was collected in 15 mL centrifugal tubes. Next, 0.25% trypsin was then added to the culture dish for digestion of transfected cells and the digested cells were collected in the tube. The tube was then vortexed and centrifuged at 1000 rpm for 10 min. The supernatant was discarded and the cell pellet was washed twice with fresh precooled PBS. The cell pellet was then resuspended in 500 μL PBS. For cell cycle analysis, the cell pellet was resuspended in 500 μL cold 75% ethanol. A flow cytometer (CytoFLEX, eBioscience, San Diego, CA, USA) was used to examine the signals for apoptosis and cell cycle based on the manufacturer’s instructions.

### Wound-healing assay

Wound healing assays were utilised to assess the migratory abilities of human trophoblast cells. Briefly, transfected JEG-3 cells were seeded in six-well plates and cultured until 90% confluent. After which, a 10 μL pipette tip was used to produce a straight wound on the cell monolayer. Subsequently, each well was rinsed twice gently using PBS and then fresh medium containing 2% FBS was added to the wells. The widths of the wounds at 0 h, 24 h and 48 h were captured using a camera attached to a light microscope. Data was analysed using Image J software (Version 1.46r).

### Transwell assay

The upper chamber of transwell inserts containing 8 µm pores (Millipore) were coated with 1 mg/mL Matrigel (BD Biosciences, Franklin Lakes, NJ, USA), which was allowed to solidify in an incubator at 37 °C for 4 h. Transfected JEG-3 cells (8 × 10^4^ cells/well) were then seeded in the upper chamber in 200 µL serum-free DMEM/F12 medium, 600 µL DMEM/F12 medium with 10% fresh FBS was added to the lower chamber. After 24 h incubation in a 5% CO_2_ atmosphere at 37 °C, invasive cells were affirmed into the matrix, whereas cells with migratory capacity passed through to the other side of the membrane, i.e., the side facing the lower chamber. Invading cells were fixed with 4% paraformaldehyde and then stained with 0.1% crystal violet (Beyotime). Invading cells were visualised using a microscope (ZEISS).

### RNA-seq analysis

Total RNA was extracted from logarithmically growing JEG3 cells that were transfected with the negative control or TOP2A-knockdown virus using Trizol (Invitrogen, Waltham, MA, USA) according to the manufacturer’s instructions. Total RNA samples were couriered to Beijing Genomics institution. Nano Drop and Agilent 2100 bioanalyzer (Thermo Fisher Scientific, Waltham, MA, USA) were used to assess the RNA quality and quantify the total RNA. To purify mRNA, magnetic beads attached with Oligo(dT) were used. Reverse transcription was performed using of random hexamer-primers to generate the first-strand cDNA followed by the synthesis of a second-strand cDNA. PCR was used to amplify the cDNA fragments. Quality controls were performed on these products with the Agilent 2100 bioanalyzer. To obtain the final library, the double stranded PCR products were subjected to heat denaturing and circularisation. Following successful establishment of the final library, the sequenced data was filtered by SOAPnuke (Version 1.5.2) and clean FASTQ format reads were obtained. Finally, differential expression analyses were performed by DESeq2 (Version 1.4.5) with Q values less than 0.05. To gain insight into the changes in phenotypes, Gene Ontology (GO) and Kyoto Encyclopaedia of Genes and Genomes (KEGG) analysis were carried out for annotation of different expressed genes using Phyper with hypergeometric tests.

### Plasmid transfection

The HTR-8/SVneo cells were transfected with either the TOP2A overexpression plasmid or the control vector. Briefly, one day before transfection, HTR-8/SVneo cells (2 × 10^5^ cells/mL) were seeded in 6-well plates. The previous medium was replaced by 900 μL of fresh medium containing 10% FBS one hour earlier before achieving 60%-70% cell confluency. The above-mentioned plasmids and PolyJet solution were diluted with serum-free medium (Cat. SL100688, SignaGen, Frederick, MD, USA) and mixed gently before being added to the 6-well plate. After 10 h, the medium was replaced with fresh medium. Two days later, mRNA or protein was extracted from the cells for use in follow-up experiments.

### Lentivirus infection

Lentiviruses loaded with short hairpin RNA coding for human TOP2A were obtained from Genechem (Shanghai, China). The sequence of TOP2A targeting short hairpin RNA (shRNA) was 5′-ATCCTGCAGGAATGGCATT-3′. Whereas the sequence for the negative control shRNA was 5′-TTCTCCGAACGTGTCACGT-3′. In preliminary experiments, the lentiviral complex infection index was determined to be 60. When JEG-3 cells reached 30–40% confluency, lentivirus transfection was performed according to the manufacturer’s instructions. After 48 h, the transfected cells were selected by culture in 2 μg/mL puromycin for seven days to identify stable clones of transfected cells. Cells were then harvested for western blot and other analyses.

### Embryo work

Kunming strain mice used in the present study were obtained from the corporation of GemPharmatech Co., Ltd. (Nanjing, China). Mice were kept in 50–70% humidity and exposed to 12/12 h dark/light cycles with ad libitum access to water and food. Protocols for animal experiments used in the present study were approved by the Animal Care Commission of Chongqing medical university.

Approximately two hundred and seventy 6–8 week old female mice were superovulated by an initial intraperitoneal injection of 10 IU pregnant mare serum gonadotropin initially, followed by 10 IU human chorionic gonadotropin 42–48 h later. Thereafter, the female mice were housed with sexually mature males in the same cage at a ratio of 1:2. Females carrying vaginal plugs were considered as 0.5 days after conception. Consistently, 1.5 days after conception, these female mice were sacrificed and their fallopian tubes were obtained under sterile conditions at 9:00.

The samples were transferred immediately to the laboratory. Two-cell phase embryos were collected by splitting the fallopian tubes by means of a stereo microscope. After washing in PBS containing 1% bovine serum albumin (BSA) (Sigma, St. Louis, MO, USA), the two-cell embryos were cultured in balanced M16 medium under 5% CO_2_ at 37 °C. Embryos were processed at the two-cell stage with PluriSIn2 (MCE, China), a TOP2A inhibitor. The concentration of PluriSIn2 was recommended by published articles. The four-cell stage of the pre-implantation embryo or the blastocyst phase were observed between 24 and 72 h and the respective developmental rate was calculated.

### Immunofluorescence staining

Processed embryos were fixed in 4% paraformaldehyde for 30 min, rinsed, and permeabilised in PBS containing 0.5% Triton X-100 for 15 min at RT. In order to block nonspecific binding, the embryos were transferred to microdroplets containing 5% BSA in PBS for 2 h under room temperature. These samples were then incubated with primary antibody at 4 °C overnight. The following day, the embryos were washed twice in PBS containing 1% BSA and then incubated with the corresponding secondary antibody for 2 h. The nuclei were then stained with Hoechst for 30 min. The embryos were then shifted to the confocal dish, and were scanned using a laser. Multiple images were captured using the confocal microscopy. Image J was used to measure the fluorescence intensity.

### RNA isolation and quantitative real-time PCR

Pooled embryos (n = 40 embryos for each sample) were dissolved using a GenElute micro-RNA extraction Kit (Sigma) in order to isolate total RNA. Total RNA of cell sample was extracted using the Steady Pure Universal RNA Extraction Kits (Accurate Biology, AG21017, Changsha, China). Total RNA was utilised for synthesis of single-stranded complementary DNA using Evo M-MLV Mix Kits (Accurate Biology, AG11728). Real-time PCR was conducted with SYBR green premix pro taq hs qPCR Kits (Accurate Biology, AG11701). The primers sequences are listed in Additional file 1: Table S1. The following PCR cycling conditions were used: 30 s at 95 °C; 40 cycles of 5 s at 95 °C and 30 s at 60 °C. Melting-curve analysis was used to check product identities. PCR was carried out in triplicates and the values of the mean threshold cycle (Ct) were normalised to *GAPDH* expression, and finally, the relative mRNA expression levels were analysed.

### Measurement of mitochondrial superoxides in embryos

To detect the production of superoxide in embryonic mitochondria, four-cell stage embryos were analysed using the MitoSox Red mitochondrial superoxide indicator (M36008; Thermo Fisher Scientific), which was dissolved in a 1:1 mixture of dimethylsulfoxide and diluted with M16 to a final working concentration of 5 μM. Embryos were incubated with the 5 μM working solution for 30 min and then stained with Hoechst under dark conditions for 30 min. Red fluorescence was measured at 510/580 nm with a confocal laser scan microscope. Reactive oxygen species (ROS) levels were obtained based on the fluorescence intensities.

### Mitochondrial membrane potential

JC-1 is widely used to assess mitochondrial membrane potential. When the mitochondrial membrane potential is high, JC-1 aggregates in the mitochondrial matrix to form polymers (J-aggregates), which can produce red fluorescence. Whereas, when the mitochondrial membrane potential is low, JC-1 does not form polymers in the mitochondrial matrix and JC-1 monomers produce green fluorescence. The distinctive distributions of green and red fluorescence can be easily analysed using fluorescence microscopy, and the red to green ratio staining was used to assess the mitochondrial membrane potential of mouse embryos. Four-cell embryos were cultured in M16 (Sigma) media with 5 μg/mL JC-1 (MCE, HY-15534) under 5% CO_2_ at 37 °C for 30 min. The embryos were then incubated with Hoechst for 20 min, washed with PBS containing 1% BSA, and observed under a laser scanning confocal microscope. The fluorescent intensities were noted. The experiment was repeated three times for analysing statistically sound data.

### Statistical analysis

Data were analysed using SPSS 19.0 (IBM, Armonk, NY, USA) and GraphPad Prism 8 software (GraphPad software Inc., San Diego, CA, USA), and were presented as the mean ± standard error of the mean. Each assay was conducted in triplicates. The comparisons between two groups were analysed with a student’s t-test. *P *values < 0.05 were considered statistically significant.

## Results

### Low expression of TOP2A in patients with recurrent spontaneous abortion

**Fig. 1:**
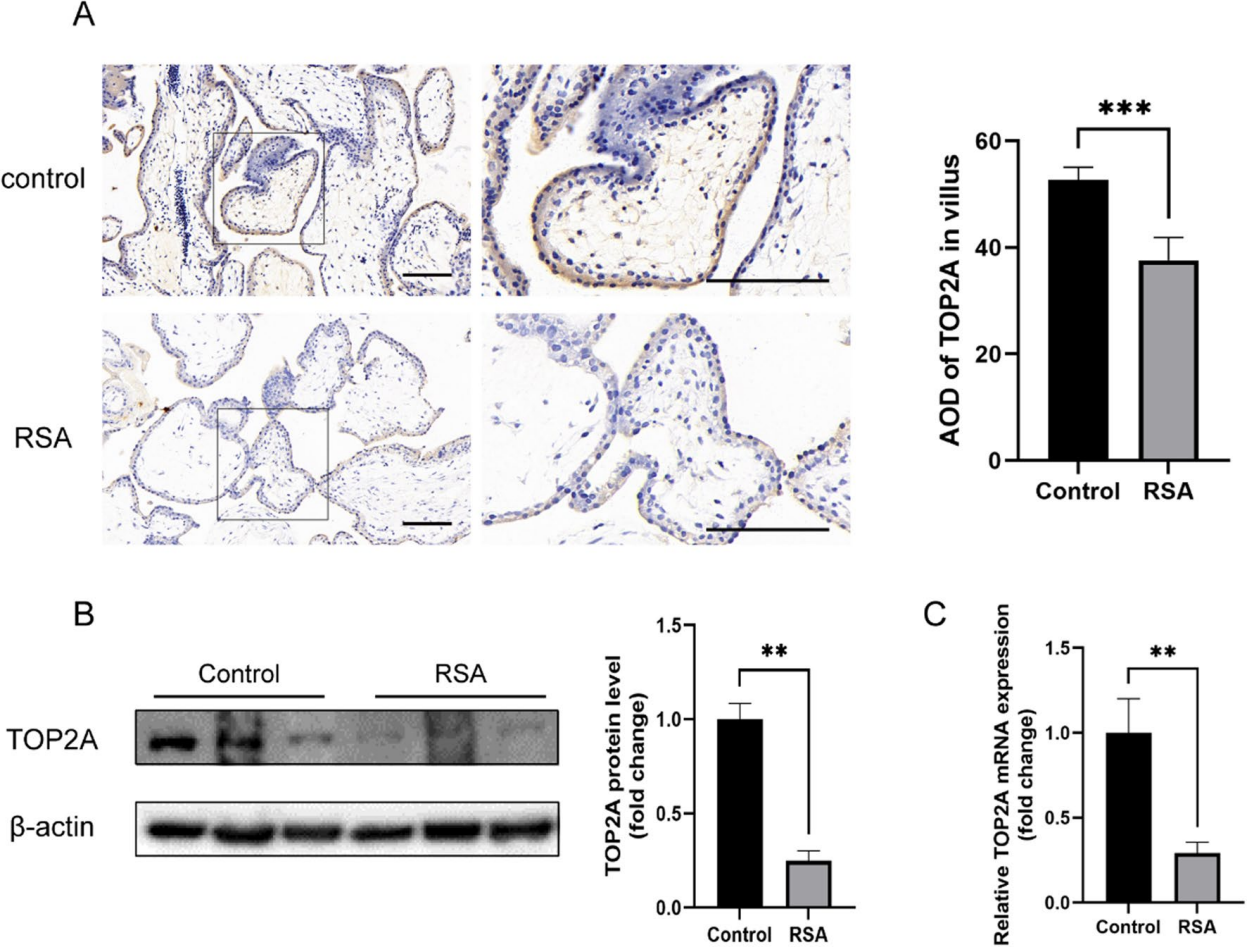
DNA Topoisomerase II Alpha (TOP2A) is expressed in low levels in villus of patients with recurrent spontaneous abortion (RSA) and is associated with RSA. **A** Immunohistochemical staining of TOP2A (*n* = 5 in each group) and relative quantitative analysis in the two groups. **B** Western blotting to assess TOP2A expression at the protein level in patients with RSA (*n* = 3). **C** *TOP2A* expression in villi from patients with RSA and the control group was analysed using qRT-PCR (*n* = 11). Student’s *t*-test was used in **A**–**C** and data are presented as the mean ± standard error of mean (SEM). **P < 0.01, ***P < 0.001. Scale bar: 100 μm

Immunohistochemistry revealed that TOP2A was localised in the nucleoplasm of villi tissues and TOP2A expression was remarkably lower in patients with RSA than that in the control group (*P* < 0.001) (Fig. 1A). Western blot analysis showed that compared with the normal group, the expression of TOP2A protein in the villi of patients with RSA decreased by 75.2% (*P* < 0.01) (Fig. 1B), which was consistent with results of qRT-PCR analysis (fold change [FC] = 0.708) (*P* < 0.01) (Fig. 1C). Collectively, these results indicate that TOP2A is expressed in the villi and that TOP2A expression is decreased in patients with RSA in comparison with that in the control group.

### TOP2A knockdown inhibits proliferation and promotes apoptosis in trophoblasts

**Fig. 2:**
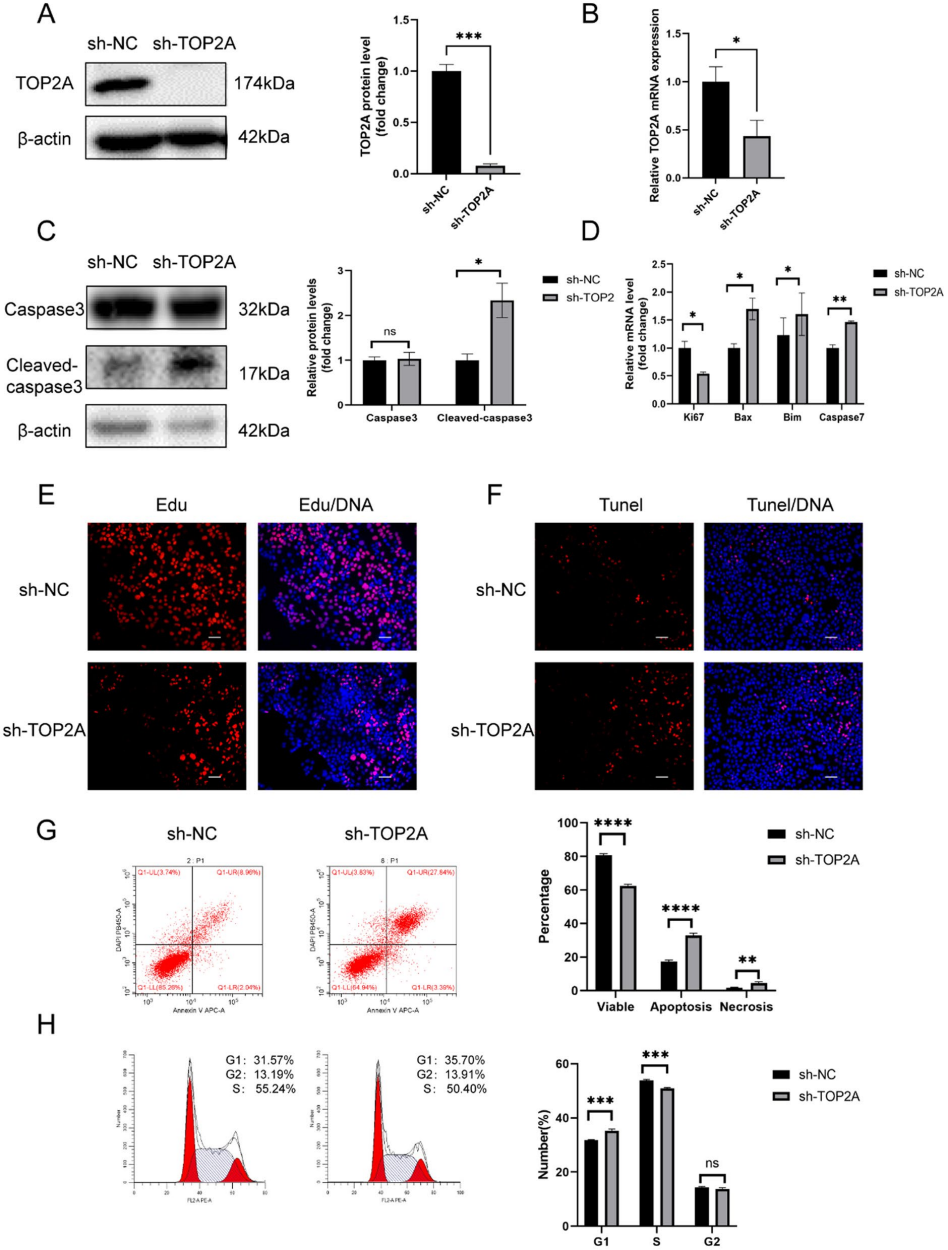
TOP2A knockdown inhibits proliferation, causes cell cycle arrest, and induces apoptosis in trophoblasts. **A** TOP2A expression was analysed after specific sh-RNA transfection. **B** mRNA levels of *TOP2A* were detected in JEG-3 cells after the indicated treatment. **C** The effect of TOP2A knockdown on the protein levels of cleaved-caspase3 and caspase3 were analysed using western blot analysis. **D** q-PCR was used to analyse the mRNA levels of *Ki67*, *Caspase7*, *Bax,* and *Bim* after TOP2A knockdown. **E** The effects of TOP2A on cell proliferation was assessed using EdU assay. **F** TUNEL assay was used to assess the effect of TOP2A knockdown on apoptosis. **G** Apoptosis was assessed via flow cytometry analysis. **H** Changes in cell cycle in treated JEG-3 cells. Each in vitro experiment was repeated three times, and data are presented as the mean ± SEM. *P < 0.05, **P < 0.01, ***P < 0.001, ****P < 0.0001. *ns* no statistical difference. Scale bar: 100 μm

**Fig. 3:**
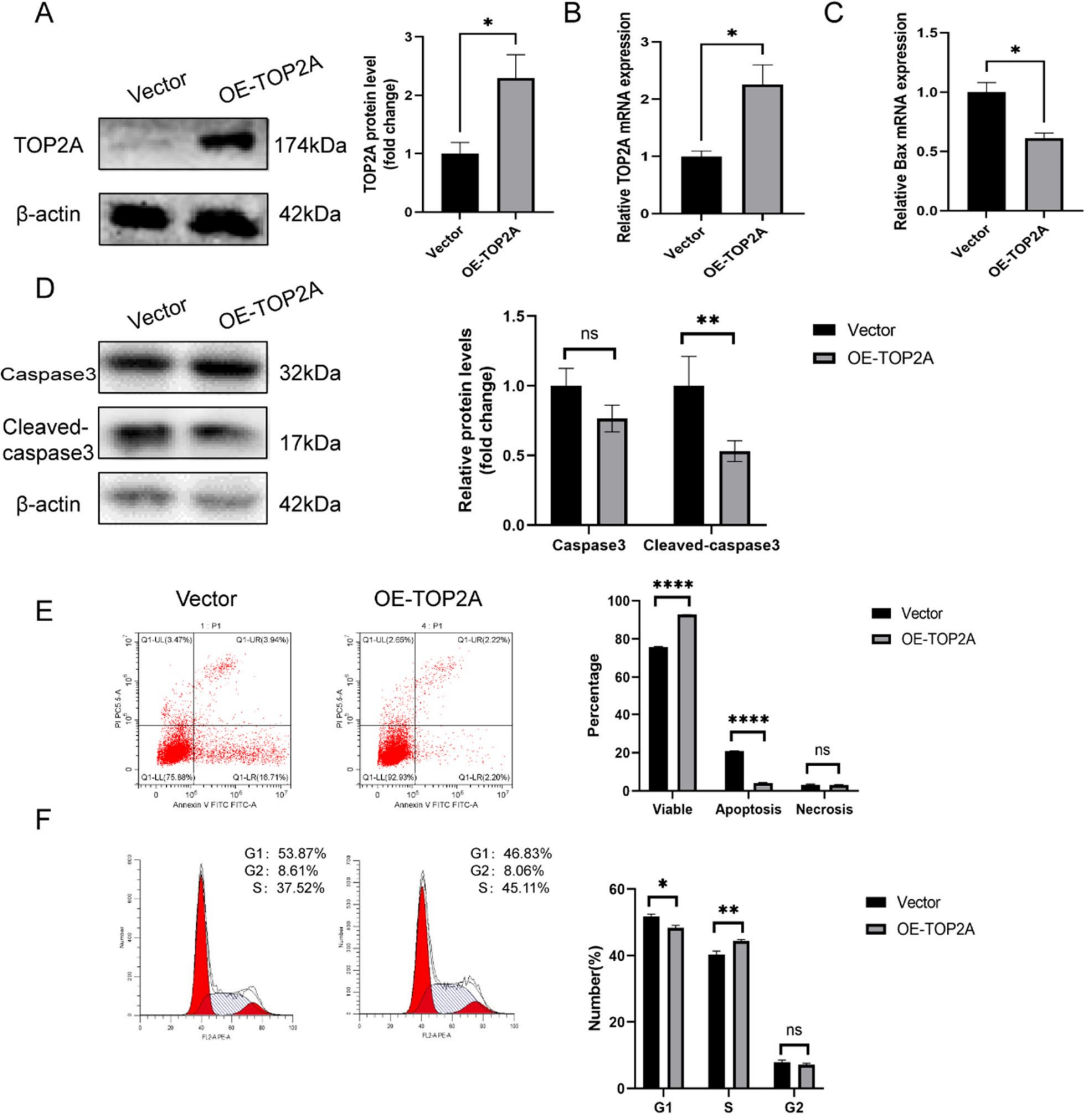
TOP2A overexpression promotes proliferation cell cycle, and inhibits apoptosis in trophoblasts. **A** Western Blot was used to test the efficiency of plasmid transfection. **B** mRNA levels of *TOP2A* were assessed in HTR-8/SVneo cells after plasmid transfection. **C** q-PCR was used to assess the mRNA level of *Bax*, an apoptosis-related gene, after TOP2A overexpression. **D** The effect of TOP2A overexpression on the protein levels of cleaved-caspase3 and caspase3 were analysed using western blot. **E** Apoptosis was assessed using flow cytometry analysis. **F** Flow cytometry analysis was used to compare differences in cell cycle in the two groups Each in vitro experiment was repeated three times, and data are presented as the mean ± SEM. *P < 0.05, **P < 0.01, ****P < 0.0001. *ns* no statistical difference

To further examine the role of TOP2A in RSA, we assessed TOP2A protein expression in four trophoblast cell lines including JEG-3, HTR8/SVneo, JAR, and BeWo. TOP2A expression was high in JEG-3 cells and low in HTR8/Svneo cells. Hence, we selected these two cell lines as in vitro models to further explore the role of TOP2A in trophoblast functions. sh-RNA targeting TOP2A and TOP2A-overexpression plasmid were transfected into to JEG-3 and Htr8 cells, respectively, to generate TOP2A knockdown (sh-TOP2A) and TOP2A-overexpression (OE-TOP2A) cells. The efficiency of knockdown and overexpression was affirmed by western blot and qRT-PCR (*P* < 0.05) (Figs. 2A, B, and 3A, B). WB analysis showed that TOP2A expression in the sh-TOP2A group decreased by 92.3% compared with that in the sh-NC group (*P* < 0.001), which was consistent with qRT-PCR analysis (FC = 0.567) (*P* < 0.05). Additionally, TOP2A protein levels in Htr8 cells in the OE-TOP2A group was 1.3 times higher than that in the vector group, which was consistent with the results of qRT-PCR (FC = 1.25) (*P* < 0.05). In terms of proliferation, EDU, a thymidine analog that is incorporated into DNA during its synthesis, was used as to assess cell proliferation. TOP2A knockdown considerably reduced the proportion of EDU-labelled cells labelled, suggesting downregulation of cell proliferation (Fig. 2E). The expression of apoptosis-related protein cleaved-caspase3 increased in sh-TOP2A JEG-3 cells (*P* < 0.05), while it decreased in OE-TOP2A HTR8/Svneo cells (*P* < 0.01). Pro-caspase 3 was not affected by knockdown or overexpression of TOP2A (Figs. 2C and 3D). qRT-PCR analysis indicated that *Ki67*, a proliferation-related gene, was down-regulated (*P* < 0.05) in the sh-TOP2A group in comparison to the sh-NC group and pro-apoptotic genes, *bim*, *bax*, and *caspase7* were up-regulated (*P* < 0.05), while the opposite was observed in the OE-TOP2A group (*P* < 0.05) (Figs. 2D and 3C). Furthermore, apoptosis ratio was also assessed with flow cytometry; overexpression of TOP2A reduced the apoptosis ratio compared to that in vector-transfected cells (*P* < 0.0001), whereas the opposite was observed in the sh-TOP2A group (*P* < 0.0001) (Figs. 2G and 3E). Fluorescence intensities in the TUNEL assay also supported these findings (Fig. 2F).

### TOP2A promotes G1-S transition in trophoblasts

We performed flow cytometry to analyse the effect of dysregulated TOP2A expression in the cell cycle (Figs. 2H and 3F). Overexpression of TOP2A in Htr8 cells resulted in a significant increase in the percentage of cells in S phase (*P* < 0.05). Whereas, downregulation of TOP2A in JEG-3 cells resulted in G1 phase arrest (*P* < 0.001). As for the G2 phase, no difference was observed in the TOP2A knockdown or plasmid-transfected groups.

### TOP2A promotes migration and invasion of trophoblast

**Fig. 4:**
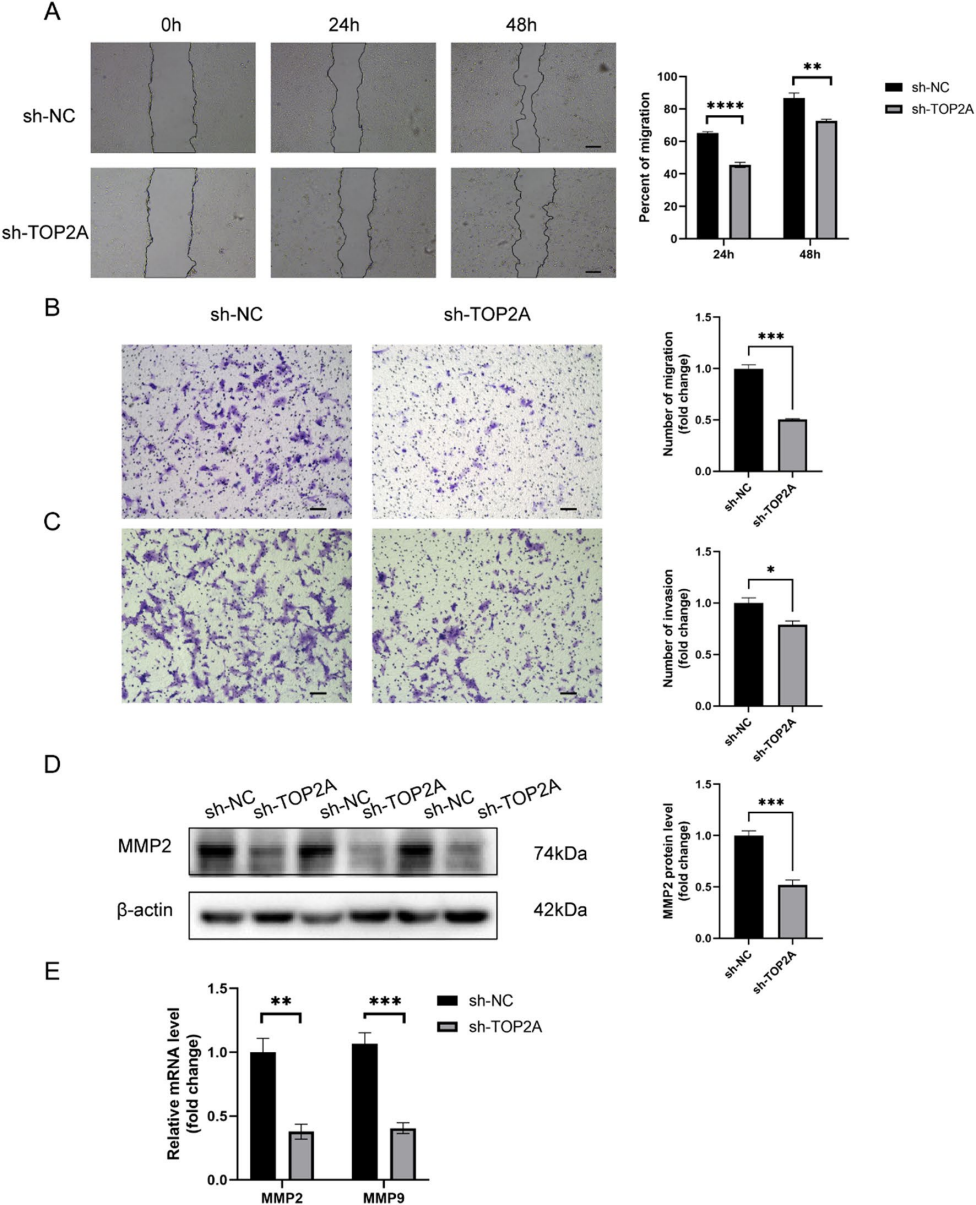
TOP2A promotes migration and invasion of trophoblast cells. **A** The effects of TOP2A knockdown on the migration rate of JEG-3 cells was determined using a wound healing assay. **B** Transwell assay was used to assess migration of trophoblasts. **C** Invasion of trophoblasts was assessed by transwell assay. **D** The effect of TOP2A on the expression of invasion-related protein metalloproteinase-2 (MMP-2) was assessed. **E** qRT-PCR was employed to determine the effect of TOP2A knockdown on the mRNA levels of *MMP2* and *MMP9*. *P < 0.05, **P < 0.01, ***P < 0.001, ****P < 0.0001. ns, no statistical difference. Scale bar: 100 μm

Transwell and wound healing assays were used to further investigate if TOP2A can influence the invasion and migration of JEG-3 cells. As shown in Fig. 4, that the number of invaded and migrated cells in the sh-TOP2A group was significantly reduced compared to that in the sh-NC group (*P* < 0.05). Real-time PCR and western blots were conducted to assess the effect of TOP2A on invasion ability. The expression of MMP-2 and MMP-9, which promote invasion, was decreased in the sh-TOP2A group compared to that in the sh-NC group (*P* < 0.01) (Fig. 4D, E). Therefore, TOP2A was determined to exert a positive effect on migration and invasion. Collectively, these findings suggest that a lack of TOP2A might cause shallow early placental implantation, which would contribute to RSA to a certain extent.

### Bioinformatic analysis of trophoblasts following TOP2A knockdown

**Fig. 5:**
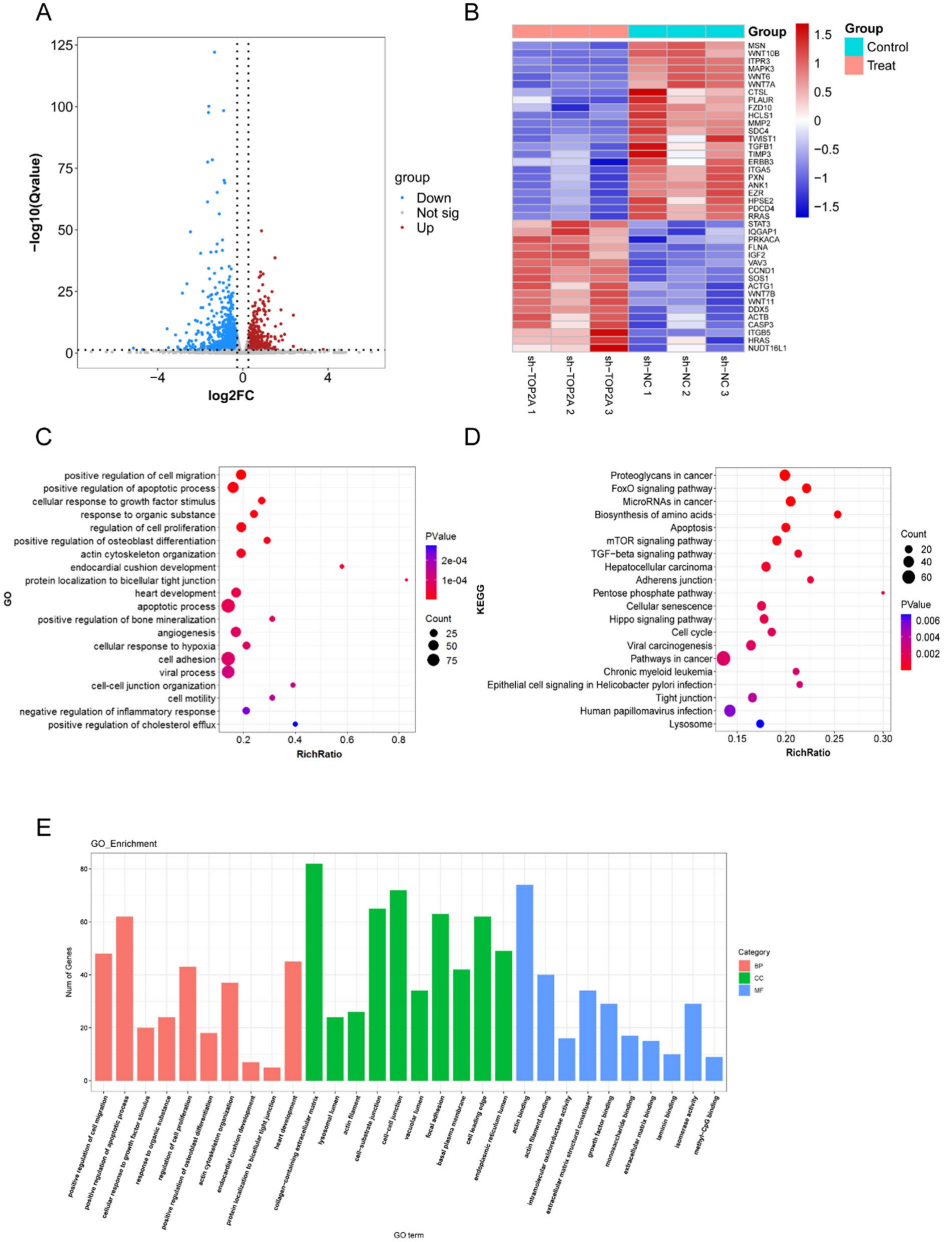
Regulatory network for TOP2A. **A**, **B** Differentially expressed genes after TOP2A knockdown are shown in volcano and heatmap plots. **C**–**E** TOP2A-associated downstream paths were illustrated using KEGG and GO enrichment analysis

The sh-NC and sh-TOP2A groups were subjected to RNA sequencing to determine alterations in TOP2A-associated genes and downstream signal transduction pathways. A total of 1764 differentially expressed genes were identified, including 798 upregulated and 966 downregulated genes (Fig. 5A, B). Genes with adjusted *P* values less than 0.05 were used to perform the enrichment analysis. KEGG analysis was used to enrich the TOP2A-associated pathways including the FOXO signalling pathway, apoptosis, and mTOR signalling pathway (Fig. 5D). Forkhead box transcription factors are involved and implicated in a broad range of cellular functions, including cellular differentiation, apoptosis, cell proliferation, DNA damage and repair, and as mediators of oxidative stress, which are critical to the biology of cancer cells (Gomes et al. 2008). Consistently, we noted a correlation between TOP2A, a vital tumour biomarker, and the FOXO signalling pathway. In addition, considering the rank of pathways that were enriched, we chose to further explore the FOXO signalling pathway. At the same time, we also further enriched the top 30 up-regulated or down-regulated genes after TOP2A knockdown (*P* < 0.05) (Additional file 1: Fig. S2).

### TOP2A promotes proliferation and cell cycle in trophoblasts via the FOXO signalling pathway

**Fig. 6:**
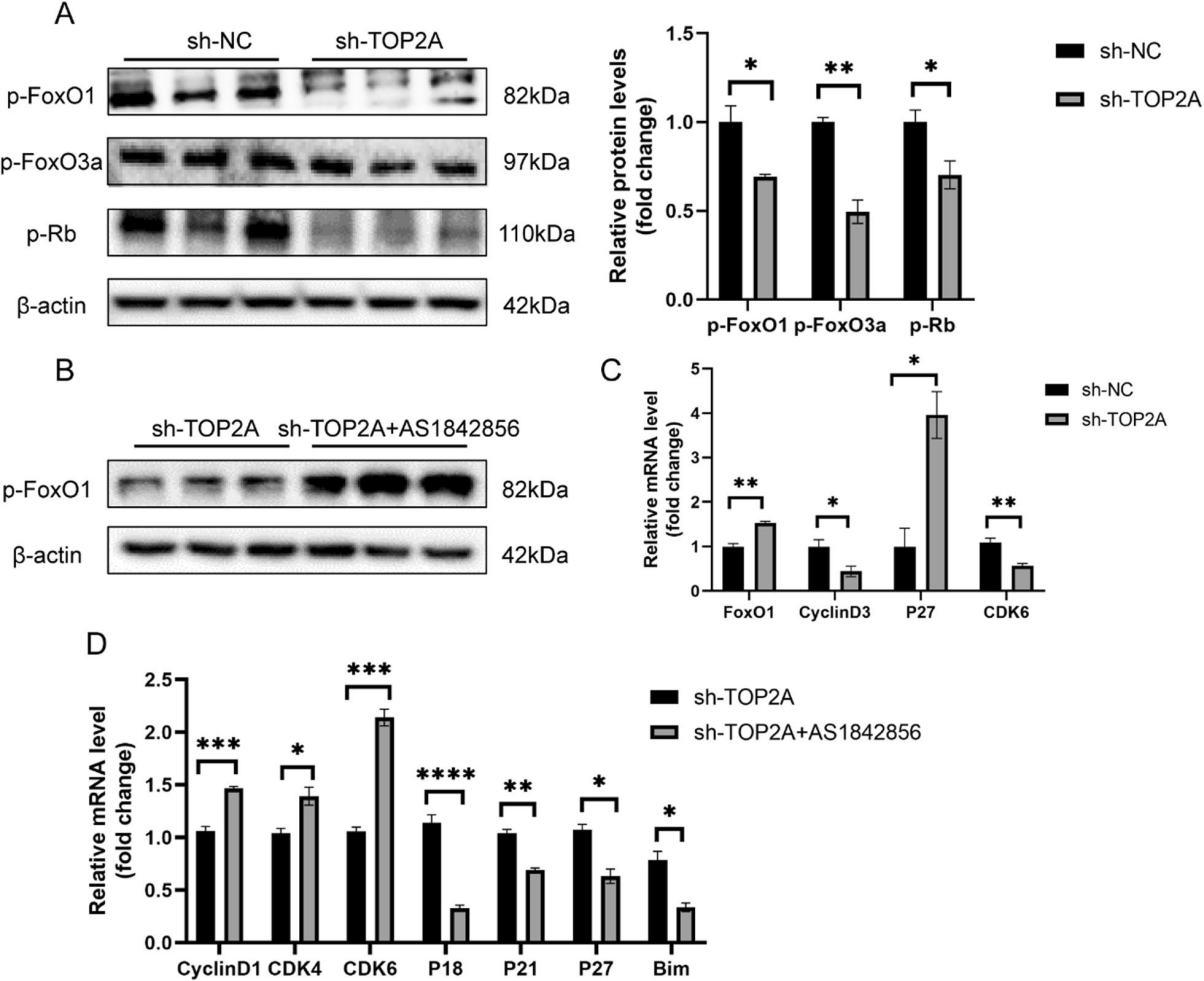
Downregulation of TOP2A affects the FOXO signaling pathway. **A** Western blotting showed changes in the levels of phosphorylated- FoxO1, phosphorylated-FoxO3a and phosphorylated-Rb after TOP2A downregulation. **B** Western blotting was used to assess changes in the level of phosphorylated-FoxO1 in the inhibitor-treated group. **C** q-PCR assay was used to verify changes in the mRNA expression of several downstream signaling molecules. **D** mRNA expression of cycle-associated genes was assessed after TOP2A knock down in JEG-3 cells and after treatment with chemical inhibitors that reduce FoxO1 activity. *P < 0.05, **P < 0.01, ***P < 0.001, ****P < 0.0001

To further investigate whether TOP2A mediates its functions via the FOXO signalling pathway, the expression of FOXO signalling pathway-related proteins and genes were measured. TOP2A knockdown reduced the levels of p-FoxO1, p-FoxO3a, and p-Rb in the sh-TOP2A group compared comparison that in the sh-NC group (*P* < 0.05) (Fig. 6A). In addition, expression of cell cycle-associated genes including *Foxo1, cyclinD3, p27,* and *CDK6* was assessed using qRT-PCR (*P* < 0.05) (Fig. 6C). The consequences obtained were in line with the forecast as well. Our data revealed that TOP2A regulates proliferation and apoptosis by regulating phosphorylation of proteins involved in the FOXO signalling pathway. To further confirm these findings, we used Htr8 cell line to verify expression of downstream signalling molecules. The expression of p‐FoxO1, p‐FoxO3a, CDK4, CDK6, and cyclinE1 was significantly upregulated in the OE-TOP2A group (*P* < 0.05) (Additional file 1: Fig. S1A) compared to that in the NC group. Consistently, qRT-PCR analysis showed that mRNA expression of *CyclinD1*, *CDK4,* and *CDK6* was upregulated in the OE-TOP2A group, where as that of *P18*, *P21*, and *P27* was significantly downregulated (*P* < 0.05) (Additional file 1: Fig. S1B). Collectively, these findings suggest that the regulatory role of TOP2A in proliferation, apoptosis, and cell cycle in JEG-3 and Htr8 cells might be associated with the FOXO signalling pathway.

### Inhibition of FOXO1 ameliorates TOP2A-knockdown associated cell cycle arrest in trophoblasts

To confirm the role of TOP2A in mediating cellular functions through its regulation of the FOXO signalling pathway, we used a specific FoxO1 inhibitor, AS1842856, to inhibit the FOXO pathway. As shown in Fig. 6B, JEG-3 cells that were subjected to TOP2A knockdown and treatment with FoxO1 inhibitor showed increased expression of p-FoxO1, which indicated the recovery of proliferation (*P* < 0.05). Likewise, These findings were consistent with results from previous studies. In addition, qRT-PCR analysis showed decreased mRNA expression of CDK inhibitors including *p18*, *p21*, *p27,* and *Bim* in the sh-TOP2A AS1842856-treated group in comparison to that in the sh-TOP2A group, while that of CDK4, CDK6, and Cyclin D1 was significantly increased (*P* < 0.05) (Fig. 6D). Consequently, suppressing the FOXO signalling pathway, to some extent, could salvage reduction of proliferation and apoptosis caused by TOP2A knockdown, which support our hypothesis.

### TOP2A inhibitor dysregulates differentiation of trophectoderm and development of mouse blastocysts

**Fig. 7:**
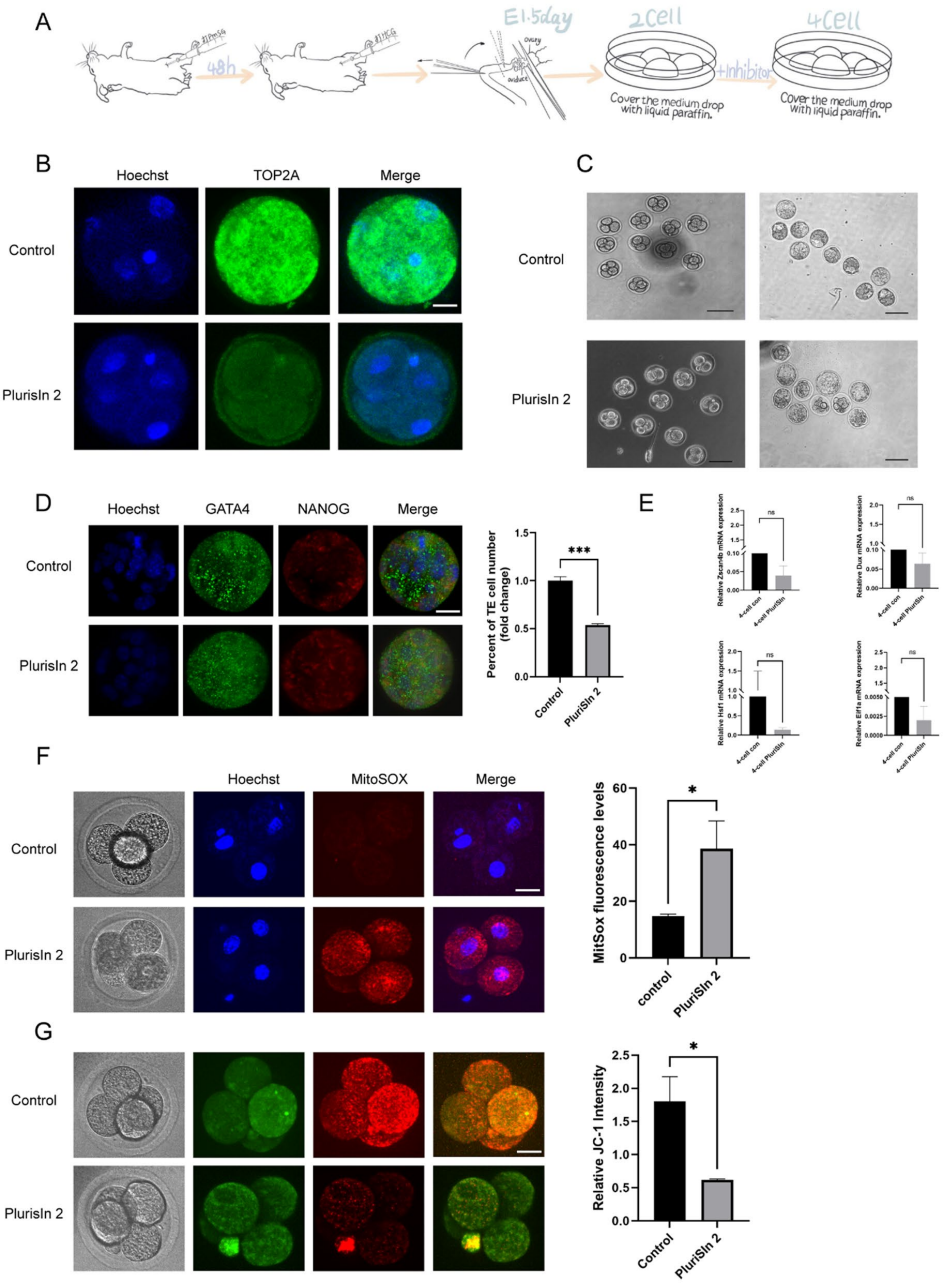
Inhibition of TOP2A affects the development potential and quality of pre-implantation embryos. **A** Flow chart depicting the protocol used for obtaining embryos. **B** Immunofluorescence was used to confirm the efficiency of the TOP2A inhibitor PluriSIn2. Total magnification: 400x. Scale bar: 20 μm **C** TOP2A inhibitor restricted the development of four-cell embryos and blastocysts. Scale bar: 100 μm. **D** Staining of nucleus/DNA (Hoechst), epiblast (NANOG), primitive endoderm (GATA4), and total ICM (NANOG and GATA4 merged). Scale bar: 20 μm **E** qRT-PCR was used to analyse the expression of the inducer of ZGA and zygotic genes to assess the effect of PluriSIn2. Total RNA was isolated from four‐cell embryos. **F** The effect of PluriSIn2 on mitochondrial superoxide levels in mouse four-cell embryos. Scale bar: 20 μm. **G** JC-1 staining of mouse four-cell embryos to determine the effect of PluriSIn2 on mitochondrial potential. Scale bar: 20 μm. *P < 0.05, ***P < 0.001. *ns* no statistical difference

The protocol used for obtaining and disposing pre-implantation embryos is depicted in Fig. 7A. To further clarify the role of TOP2A in the development of mouse blastocysts and trophectoderm differentiation, we cultured two-cell embryos in media containing PluriSIn, which is a selective TOP2A inhibitor. We confirmed that the four-cell rate and blastocyst developmental rate were notably decreased in the PluriSIn2-treated group (Fig. 7C). All developmental rates were shown in Table 2. Based on detectable and exclusive staining of NANOG and GATA4, total cell counts from DAPI staining were categorised as those corresponding to cells of the c inner cell mass or the trophectoderm (TE); TE, which are the outer cells that are not considered as a part of the inner cell mass of the blastocyst do not exhibit GATA4 and NANOG staining. As is shown in Fig. 7D (*P* < 0.001), the PluriSIn2-treated group exhibited lower TE differentiation than that of the control group.

**Table 2:** Effect of TOP2A-inhibitor on mouse preimplantation embryo development

Group	Developmental rate of four-cell(%)	Developmental rate of blastocysts(%)
M16	85.34	56.89
TOP2A-inhibitor	59.60	34.46
*P*	< 0.001	< 0.001

### TOP2A inhibitor promotes ROS accumulation in four-cell embryos

Downregulation of zygotic genome activation (ZGA)-related genes is reportedly associated with early mouse embryonic developmental block (Shi et al. 2018). Hence, we performed qRT-PCR analysis to assess whether PluriSIn2, a selective TOP2A inhibitor, can decrease embryo quality by downregulating ZGA-associated genes. As shown in Fig. 7E, no statistical difference was observed with respect to mRNA expression of zygotic genes such as *SCAN4B* (Zscan4b), eukaryotic translation initiation factor 1A (*Eif1a*), heat shock factor 1, zinc finger, and *Dux* gene, which promotes ZGA, in either the treated or untreated groups.

Next, we investigated whether inhibition of TOP2A resulted in accumulation of ROS, which in turn caused embryonic development retardation. To explore the mechanism of action of PluriSIn2-mediated TOP2A inhibition, mitochondrial superoxide, the index reflecting the level of ROS, were examined in embryos using a confocal laser-scanning microscope. As shown in Fig. 7F, mitochondrial superoxide levels in four-cell embryos treated with PluriSIn2 were significantly higher than that in the control embryos (*P* < 0.05). Given that ROS accumulation can damage the permeability of the mitochondrial membrane, we dyed JC-1 to compare the fluorescent intensity in the PluriSIn2-treated and untreated groups. Embryos incubated with the PluriSIn2 showed a significant increase green fluorescence compared to that in the control group, which suggests a decrease in mitochondrial membrane potential (*P* < 0.05) (Fig. 7G).

## Discussion

Trophoblast invasion into the uterus decidua is a critical requirement for the establishment of early pregnancy. The *TOP2A* gene encoding DNA topoisomerase II Alpha plays a role in the control and alteration of DNA topology during transcription (Tarpgaard et al. 2016). Additionally, TOP2A is a risk factor for poor survival and a reliable prognostic biomarker in different cancers to predict cancer progression and relapse (Guo et al. 2020; Kou et al. 2020; Schaefer-Klein et al. 2015; Chen et al. 2021). Given the perspective that the process of embryonic implantation is similar to tumorigenesis, we put forward the hypothesis that TOP2A plays an important role in RSA.

In the present study, we investigated whether TOP2A is involved in the pathogenesis of RSA by regulation the functions of trophoblast cells and/or development of embryos. To the best of our knowledge, we are the first to demonstrate that TOP2A expression is lower in the villi of patients with RSA. Subsequently, we chose an appropriate cell model to investigate the role of TOP2A in trophoblasts. Previously, Wang et al. reported that TOP2A was highly expressed in HCC and had a positive effect on cancer cell proliferation as well as invasion (Wang et al. 2022). In addition, Zhou et al. reported that repressing TOP2A would damage the phenotypes in the tumour cell lines (Zhou et al. 2021b). Consistent with findings of previous studies in tumours, in the present study, we observed that TOP2A knockdown negatively effects trophoblast functions including proliferation, apoptosis, migration, and invasion. To further understand the underlying mechanism, RNA-seq and KEGG enrichment analysis were carried out. It is well-known that Forkhead box transcription factors are involved in the regulation of a broad range of cellular functions, including cellular differentiation, apoptosis, cell proliferation, DNA damage and repair, and oxidative stress, all critical to the cell biology of cancer (Gomes et al. 2008). Taking this into consideration, in the present study, we concentrated on the FOXO signalling pathway. In the presence of growth factors, FOXO is translocated to the cytosol, where it is subjected to degradation through the ubiquitin–proteasome pathway. Whereas, in the absence of growth factors, FOXO translocated to the nucleus and activates target genes, thereby inducing cell cycle arrest, apoptosis, and resistance to stress (Greer and Brunet 2005). Here, we observed that phosphorylated -FoxO1a and FoxO3a were significantly decreased following TOP2A knockdown, suggesting that FOXO signalling pathway may be activated in RSA and give rise to the abnormal functions of trophoblast that take part in early spontaneous abortion (Hustin et al. 1990).

To further verify that the effect of TOP2A on the development and quality of pre-implantation embryos, given its effect on trophoblast functions, we used PluriSIn2, a TOP2A inhibitor. Two-cell embryos were cultured in M16 medium containing PluriSIn2 to observe the effect of TOP2A inhibition on the developmental rate of four-cell embryos and blastocysts. We observed that embryos treated with PluriSIn2 showed a reduced developmental rate compared to the control group, indicating that TOP2A inhibition negatively effects embryo quality. Furthermore, pooled embryos showed decreased differentiation of TE than that of the control group, suggesting that TOP2A may hinder the growth and functions of TE, thereby contributing to RSA.

Subsequently, we focused on the underlying mechanism of TOP2A inhibition mediated damage of developmental potential in early embryos.

ZGA refers to the initial production of zygotic mRNAs transcribed from the zygotic genome at a species-specific stage, which replace maternal transcripts and play a part in early embryogenesis before implantation. Mammalian embryos that cannot regulate proper activation of embryonic genome could stop development (Schultz 1993; Minami et al. 2007). In view of the importance of ZGA, we aimed to investigate whether TOP2A affects this event in the developmental process. However, there was no difference in the expression of zygotic genes nor in the induction of ZGA, suggesting that TOP2A does not play a role in this process.

In oocytes and early embryos, mitochondria play a pivotal role in ATP generation as mitochondrial-based oxidative metabolism is required for development, rather than glycolysis (Houghton et al. 1996). The main function of mitochondria is ATP generation, which is directly associated with the potential of embryo development (Blerkom et al. 1995). Apart from increased rates of aneuploidy, mitochondrial dysfunctions, including oxidative damage, changes in mitochondrial membrane potential, and decreased ATP generation, are observed in oocytes and embryos of older mothers, compared with that of younger counterparts in both human and animal models (Wilding et al. 2001; Wilding et al. 2003; Hamatani et al. 2004; Eichenlaub-Ritter et al. 2011; Tatone et al. 2011), indicating that reduced rates of blastocyst development can be partly attributed to metabolic dysfunction. Therefore, in the present study, we examined mitochondrial superoxide levels in embryos and found that levels increase in the TOP2A inhibitor-treated group as compared to the control group, suggesting that inhibition of TOP2A impairs mitochondrial functions. Consistently, we observed decreased mitochondrial membrane potential following inhibition of TOP2A.In addition to the role of TOP2A in pre-implantation embryos, we explored the role of TOP2A in cells of the TE of blastocysts.

Collectively, we observed that inhibition of TOP2A can suppress trophoblast proliferation, invasion, and migration via the FOXO signalling pathway and impair the development potential of pre-implantation embryos by dysregulating mitochondrial functions and not ZGA.

The present study has some limitations. The experimental time period was relatively short, and the collected samples were limited by regions. Therefore, in the future, we plan to increase the sample size by including patients from multiple centres. We also plan to investigate the specific underlying mechanism through which TOP2A participates in the pathogenesis of RSA by using animal models.

## Conclusions

In summary, we observed that knockdown of TOP2A not only inhibited the proliferation, migration and invasion of trophoblasts, which was determined by regulation of the FOXO signalling pathway, but also impaired the growth and development of mouse preimplantation embryos via excessive oxidative stress, which could further cause RSA. These results suggest that a role of TOP2A in regulating the functions of trophoblasts and development of pre-implantation embryos in mice, suggesting that TOP2A is a potential biomarker and a therapeutic target for RSA.

## Supplementary Information

**Additional file 1: Table S1.** Sequence information of the PCR primers. **Figure S1.** Overexpression of TOP2A promotes cell cycle via the FoxO signalling pathway. **Figure S2**. GO analysis of up-regulated and down-regulated differential genes

## Data Availability

The datasets used and/or analysed during the current study are available from the corresponding author on reasonable request.

## Abbreviations

BSA: Bovine serum albumin
CDK: Cyclin-dependent kinase
EVT: Extravillous trophoblast
FC: Fold change
GO: Gene ontology
HCC: Hepatocellular carcinoma
KEGG: Kyoto Encyclopedia of Genes and Genomes
MMP: Matrix metalloproteinase
OE-TOP2A: TOP2A-overexpression
PBS: Phosphate-buffered saline
ROS: Reactive oxygen species
RSA: Recurrent spontaneous abortion
sh-TOP2A: TOP2A knockdown
TE: trophectoderm
TOP2A: DNA Topoisomerase II Alpha
ZGA: Zygotic genome activation
