# Supplementary material for: TOP2A deficiency leads to human recurrent spontaneous abortion and growth retardation of mouse pre-implantation embryos
Source: Mol Med. 2022 Dec 30;28:165. doi: 10.1186/s10020-022-00592-4 (PMC9805267; doi:10.1186/s10020-022-00592-4)
Supplement: Supplementary file 1 — Additional file 1: Table S1. Sequence information of the PCR primers. Figure S1. Overexpression of TOP2A promotes cell cycle via the FoxO signalling pathway. Figure S2. GO analysis of up-regulated and down-regulated differential genes [file 10020_2022_592_MOESM1_ESM.docx]

**Additional file 1: Table S1.** Sequence information of the PCR primers.

| Gene Symbol | Primer sequences |
| --- | --- |
| Zscan4b-F | GCCACAAGACCAACCTGAAGTA |
| Zscan4b-R | GCGAAAGCGACGGCTACATA |
| Hsf1-F | GCAGAGAACAGTAACCCCGA |
| Hsf1-R | CATCCGTGTAGTCATCCCCC |
| Eif1a-F | ACTCCTGTGGTTGCCCTTTC |
| Eif1a-R | GCATACTCCTGCCCATCCTC |
| Dux-F | AGGAAGACGGTTTGGCAG |
| Dux-R | TGAGACCCCCATTCGCTT |
| MMP9-F | CGGACCAAGGATACAGTTTGTT |
| MMP9-R | TCAGGGCGAGGACCATAGA |
| MMP2-F | CCCTCCCTTCAACCATTCCC |
| MMP2-R | TTCCAGCAGACACCATCACC |
| MKI67-F | TTTGGGTGCGACTTGACGA |
| MKI67-R | TCCTTTTGATAGTAACCAGGCGT |
| BAX-F | TCCTCTCCTACTTTGGGA |
| BAX-R | CTTCTTCCAGATGGTGAG |
| CASP7-F | ATCAATGACACAGATGCTAATC |
| CASP7-R | CTGGAACCGTGGAATAGG |
| top2a-F | GGGCACCAGCACATCAAAG |
| top2a-R | TAGCAGCATCATCTTCAGGACC |
| CDK4-F | ATTGGTGTCGGTGCCTATGG |
| CDK4-R | ACGAACTGTGCTGATGGGAA |
| CDK6-F | CCTTAGCACAGCACCACAGA |
| CDK6-R | AATGAGGCGGGGGATTTCTC |
| p18-F | ACAGACTTTGCTGGAGTTTCAAG |
| p18-R | TTATGGTTCCGATGCCCCAC |
| cyclinD3-F | CCTCTCCCATTGTCCCTCTG |
| cyclinD3-R | GCCACCAGCCTAAACCTTG |
| P21-F | CCCTTGTCCTTTCCCTTCAGT |
| P21-R | CCCTTCTTCTTGTGTGTCCCT |
| P27-F | CGGGACTTGGAGAAGCACT |
| P27-R | GGTCTGTAGTAGAACTCGGGC |
| CyclinD1-F | CACGCTTACCTCAACCATCCT |
| CyclinD1-R | GCCCCATCACGACAGACAA |
| Foxo1-F | AGGTGGAGGTTGGTTTTGTAGT |
| Foxo1-R | CAGGGCAGAAGGGAGAATGAG |
| BIM-F | TGATGTAAGTTCTGAGTGTGACCG |
| BIM-R | CTTGTGGCTCTGTCTGTAGGG |


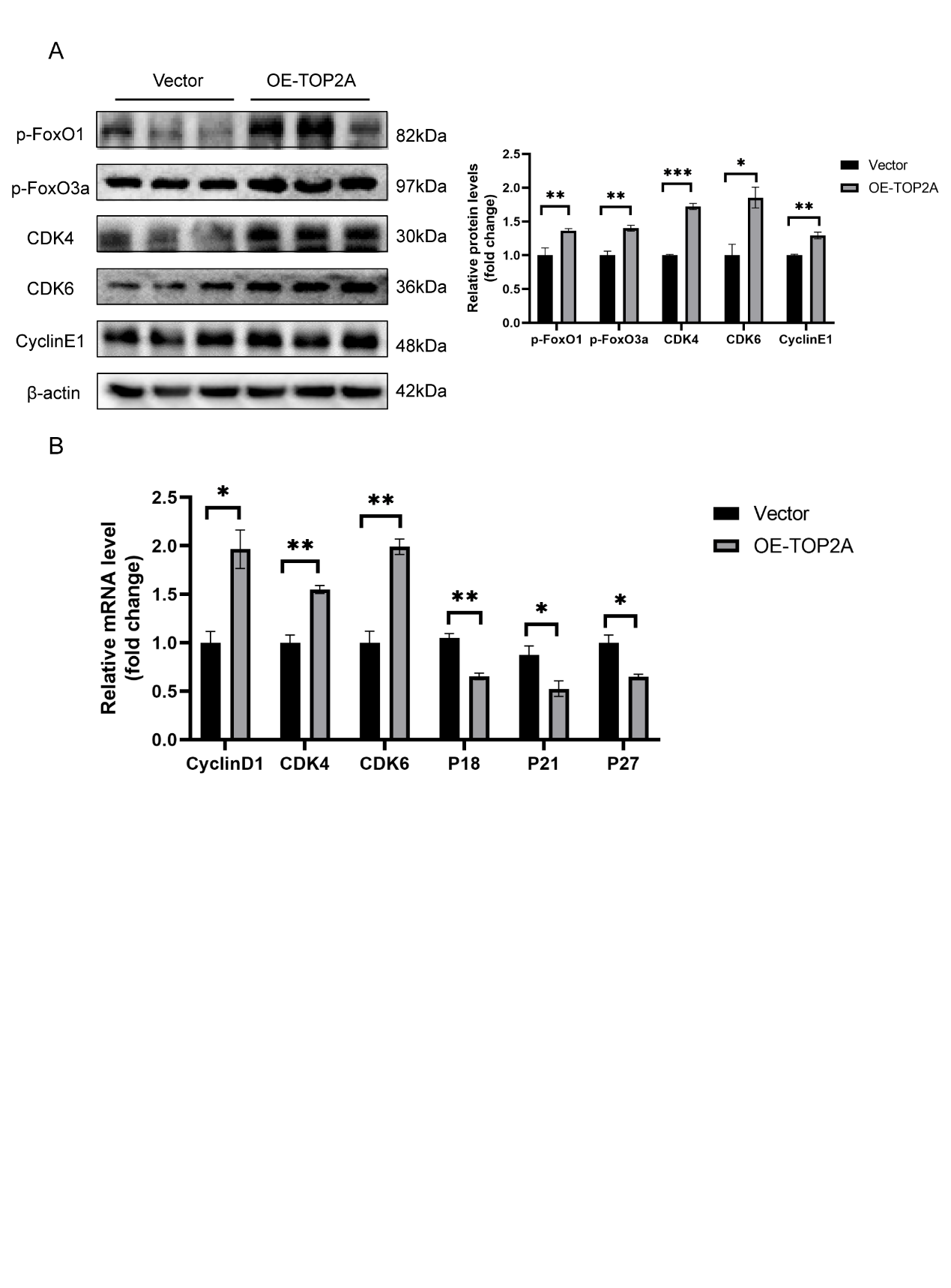


**Additional file 1: Figure S1**


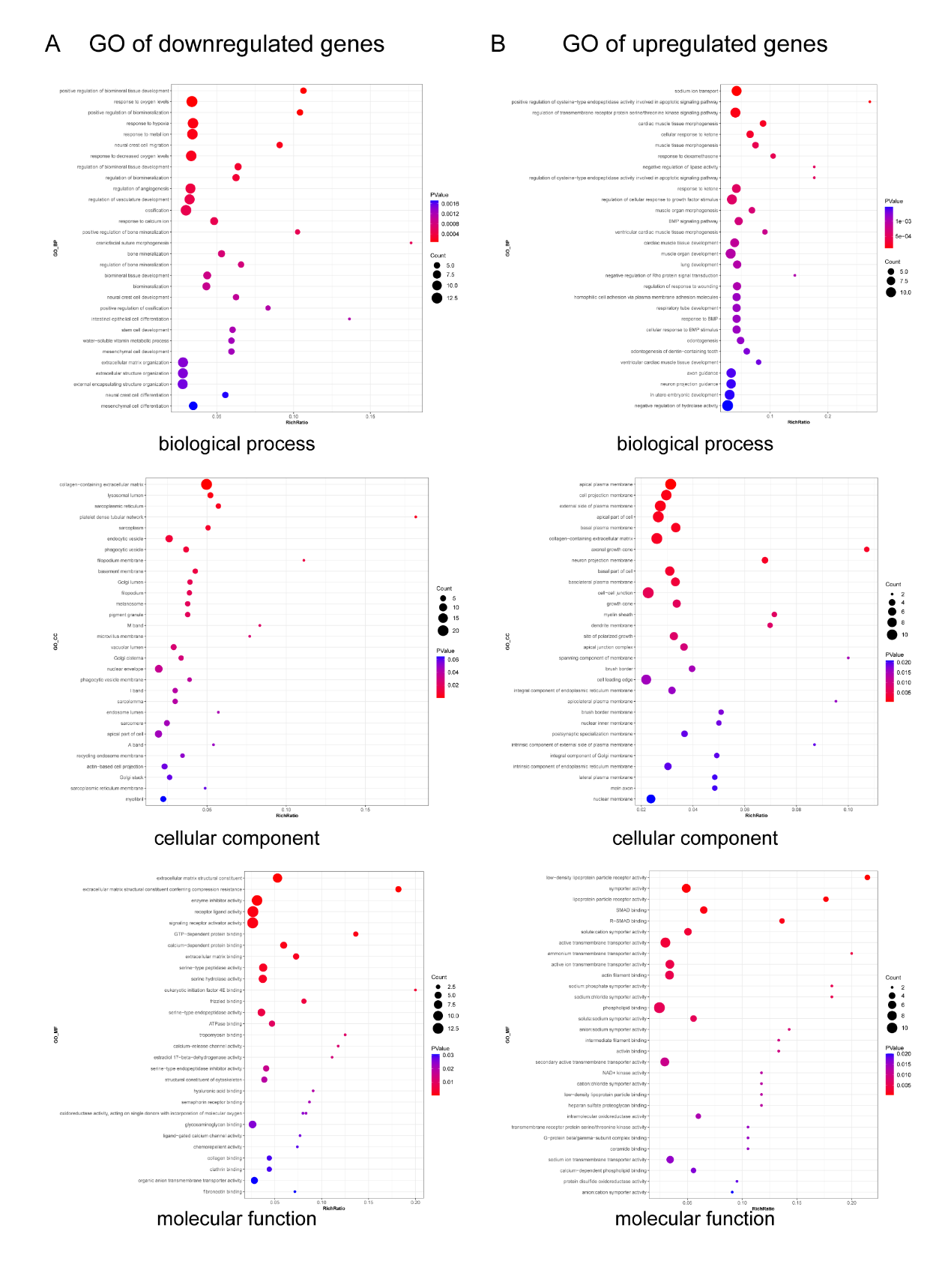


**Additional file 1: Figure S2**

**Additional file 1: Material**

Additional file 1: Figures

Additional file 1: Figure S1 Overexpression of TOP2A promotes cell cycle via the FoxO signalling pathway.

(A) Western blot analysis of proteins involved in the FoxO signalling pathway in HTR-8/SVneo cells. (B) qRT-PCR analysis of FoxO signalling pathway-related genes (such as *CyclinD1, CDK4, CDK6, P18, P21,* and *P27*) in vector control and OE-TOP2A cells. **P* < 0.05, ***P* < 0.01, ****P* < 0.001.

Additional file 1: Figure S2 GO analysis of up-regulated and down-regulated differential genes

(A) GO analysis of the top 30 differential genes downregulated after TOP2A knockdown. (B) GO Analysis of the top 30 differential genes upregulated after TOP2A knockdown.
